# An Oxidized Mogrol Derivative Alleviates LPS-Induced Acute Lung Injury in Association with Reduced NLRP3 Inflammasome-Associated Inflammatory Responses in Mice

**DOI:** 10.3390/ijms27219584

**Published:** 2026-10-27

**Authors:** Guizeng Feng, Yinting Tuo, Jian Su, Feng Huang, Dianpeng Li, Lini Huo, Xing Xia

**Affiliations:** 1 College of Pharmacy, Guangxi University of Chinese Medicine, Nanning 530200, China; fengguizeng2024@stu.gxtcmu.edu.cn (G.F.); tuoyinting2023@stu.gxtcmu.edu.cn (Y.T.); sujian2023@stu.gxtcmu.edu.cn (J.S.); huangfeng2023@stu.gxtcmu.edu.cn (F.H.); ldp@gxib.cn (D.L.); 2 Guangxi Academy of Sciences, Nanning 530007, China; 3 Guangxi Engineering Research Center for High-Value Utilization of Guangxi-Produced Authentic Medicinal Herbs, Guangxi University of Chinese Medicine, Nanning 530200, China

**Keywords:** mogrol, oxidative derivatives, acute lung injury, NLRP3 inflammasome

## Abstract

Acute lung injury (ALI) is a severe inflammatory pulmonary disorder with high morbidity and limited treatment options available in clinical settings. Mogrosides, natural products derived from *Siraitia grosvenorii*, have been shown to exhibit protective effects against ALI, but structural optimization of their aglycone moiety, mogrol, may provide opportunities to improve pharmacological efficacy. In this study, oxidized mogrol derivatives (MG-COOH and MG-CHO) were synthesized, and their anti-inflammatory activities were initially compared with those of natural mogrosides in lipopolysaccharide (LPS)-stimulated MH-S cells. MG-COOH exhibited pronounced anti-inflammatory activity and was designated for comprehensive analysis of its protective potential and underlying mechanisms in the LPS-induced ALI model. MG-COOH effectively alleviated LPS-induced injury in lung, decreased pro-inflammatory cytokine release, and reduced oxidative stress, showing a tendency toward greater efficacy relative to natural mogrosides at equimolar doses. Transcriptomic analysis indicated that MG-COOH modulated genes involved in the HIF-1, JAK-STAT, MAPK, PI3K-Akt, NF-κB, NOD-like receptor, and Toll-like receptor signaling pathways. Furthermore, MG-COOH reduced the expression of NLRP3, ASC, caspase-1, and IL-1β mRNA and protein in lung. In conclusion, the oxidized mogrol derivative MG-COOH exhibits pronounced anti-ALI activity, which is associated with reduced NLRP3 inflammasome-related inflammatory responses in the lung, which serves as a pivotal node in multiple inflammatory signaling cascades.

## 1. Introduction

ALI is a clinical syndrome triggered by a variety of harmful factors, ranging from acute pneumonia and sepsis to severe trauma, with severe cases possibly developing acute respiratory failure [1,2]. An estimated 200,000 cases of ALI are reported each year in the United States, with a mortality rate reaching approximately 40% [3,4]. Glucocorticoids and other anti-inflammatory agents are commonly employed for the management of ALI in clinical settings. While these therapies may alleviate inflammatory symptoms and clinical manifestations, their therapeutic value is limited due to the development of treatment resistance and adverse reactions. Accordingly, it is critically important to create new functional substances capable of providing protection against ALI.

Inflammatory response and oxidative stress are key pathogenic mechanisms of ALI [5]. These two processes are interconnected through a self-amplifying cycle: inflammatory responses promote oxidative stress, the resulting excessive accumulation of reactive oxygen species (ROS) further intensifies inflammation, and both contribute to the progression of ALI across its pathophysiological development. Exposure to LPS induces ALI via a dual mechanism. In addition to directly triggering inflammatory signaling, LPS disrupts pulmonary redox homeostasis, which drives an abnormal accumulation of ROS within lung tissues. The oxidative burden exacerbates pulmonary edema and inflammatory cell infiltration, and sparks a robust outflow of pro-inflammatory cytokines and chemokines, eventually leading to ALI [6,7,8]. Numerous studies have shown that the MAPK, PI3K-Akt, TNF, JAK-STAT, and NF-κB signaling pathways play a vital role in both the pathogenesis of inflammatory injury and the lung-protective actions of bioactive agents against ALI [9,10,11,12]. Notably, most of these pathways modulate NLRP3 inflammasome activation via direct or indirect transcriptional regulation. Accumulating evidence indicates that the attenuation of NLRP3 inflammasome-related signaling and reduced inflammatory factor release contribute to the alleviation of LPS-induced ALI [13].

*Siraitia grosvenorii*, a herb long employed in traditional Chinese medicine, is valued for its functions: relieving cough, reducing inflammation, and regulating glucose metabolism [14,15]. The principal active components of *Siraitia grosvenorii* are cucurbitane-type tetracyclic triterpenoid saponins, such as mogroside V (MG-V). Upon oral administration, these saponins undergo sequential deglycosylation mostly in the intestinal tract, yielding lower glycoside metabolites such as mogroside IIIA (MG-IIIA), mogroside IIA (MG-IIA), and mogroside IE (MG-IE), which are further metabolized to the aglycone mogrol. Only a small amount of mogrol and low-glycoside saponins can be absorbed into the blood circulation. Accumulating evidence demonstrates that mogrosides and their deglycosylated metabolites attenuate oxidative stress and inflammation, and exhibit potent protective effects against lung injury [8,10,16,17,18]. In particular, mogrol can suppress inflammatory responses in RAW264.7 cells and ameliorate bleomycin-induced pulmonary fibrosis [19,20]. The studies on the anti-inflammatory activity of triterpenoids have revealed that the presence of a free carboxyl group on the carbon skeleton is a key determinant of their anti-inflammatory efficacy [21]. A variety of triterpenic acids with carboxyl side chains, such as glycyrrhetinic acid, oleanolic acid and ursolic acid, have been confirmed to exert pronounced anti-inflammatory activities [22,23]. As a triterpenoid compound, mogrol may exhibit improved anti-inflammatory activity after carboxyl group modification. Mogrosides and their aglycone mogrol exhibit favorable safety profiles and protective effects against pulmonary inflammatory disorders [24]. Structural modification of their side chains may afford additional mogrosides with high bioactivity and broaden clinical application potential. Hence, this strategy may facilitate the discovery of more effective small-molecule candidates for the prevention of inflammatory pulmonary diseases.

In this study, we chemically synthesized carboxyl-modified mogrol derivatives and compared their anti-inflammatory activities with those of natural mogrosides. Based on RNA sequencing (RNA-Seq) analysis, we explored the anti-ALI mechanism of the derivative, which appears to involve the attenuation of NLRP3 inflammasome-related signaling.

## 2. Results

### 2.1. Synthesis of MG-CHO and MG-COOH

**Figure 1:**
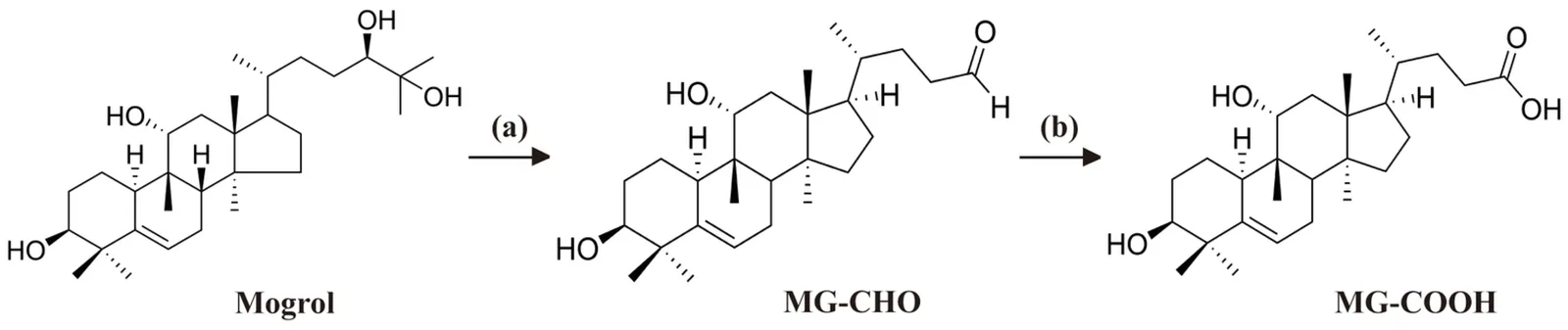
Synthetic scheme for the oxidized mogrol derivatives, MG-CHO and MG-COOH. Reagents and conditions: (**a**) NaIO_4_, acetone/H_2_O, 60 °C, 20 h. (**b**) NaClO_2_, 2-methyl-2-butene, t-BuOH/NaH_2_PO_4_/H_2_O, rt, 20 h.

The oxidized mogrol derivatives were synthesized following a previously reported procedure [25,26], with further optimization, and the synthetic routes are illustrated in Figure 1. Using mogrol as the starting material, the intermediate MG-CHO was prepared via selective oxidation of the vicinal diol moiety with sodium periodate, which was then converted to MG-COOH through Pinnick oxidation of the aldehyde group to the corresponding carboxylic acid. Owing to the significantly enhanced polarity of both oxidized products relative to mogrol, their solubility in the aqueous phase was substantially reduced. Consequently, MG-CHO could be directly precipitated from the reaction mixture upon the addition of water and standing, while MG-COOH crystallized from saturated ammonium chloride solution after standing for 2 h. In terms of yields, MG-CHO was obtained in 78.5% yield, slightly higher than the reported 77%. The yield of MG-COOH was consistent with the literature procedure, affording the product in over 90% yield. The simplified crystallization post-treatment omits steps such as extraction, drying, concentration, and column chromatography purification found in traditional methods. It can complete product purification without compromising yield or purity, significantly enhancing overall synthetic efficiency.

### 2.2. Effects of MG-COOH, MG-CHO, and Mogrosides on MH-S Cells Viability

**Figure 2:**
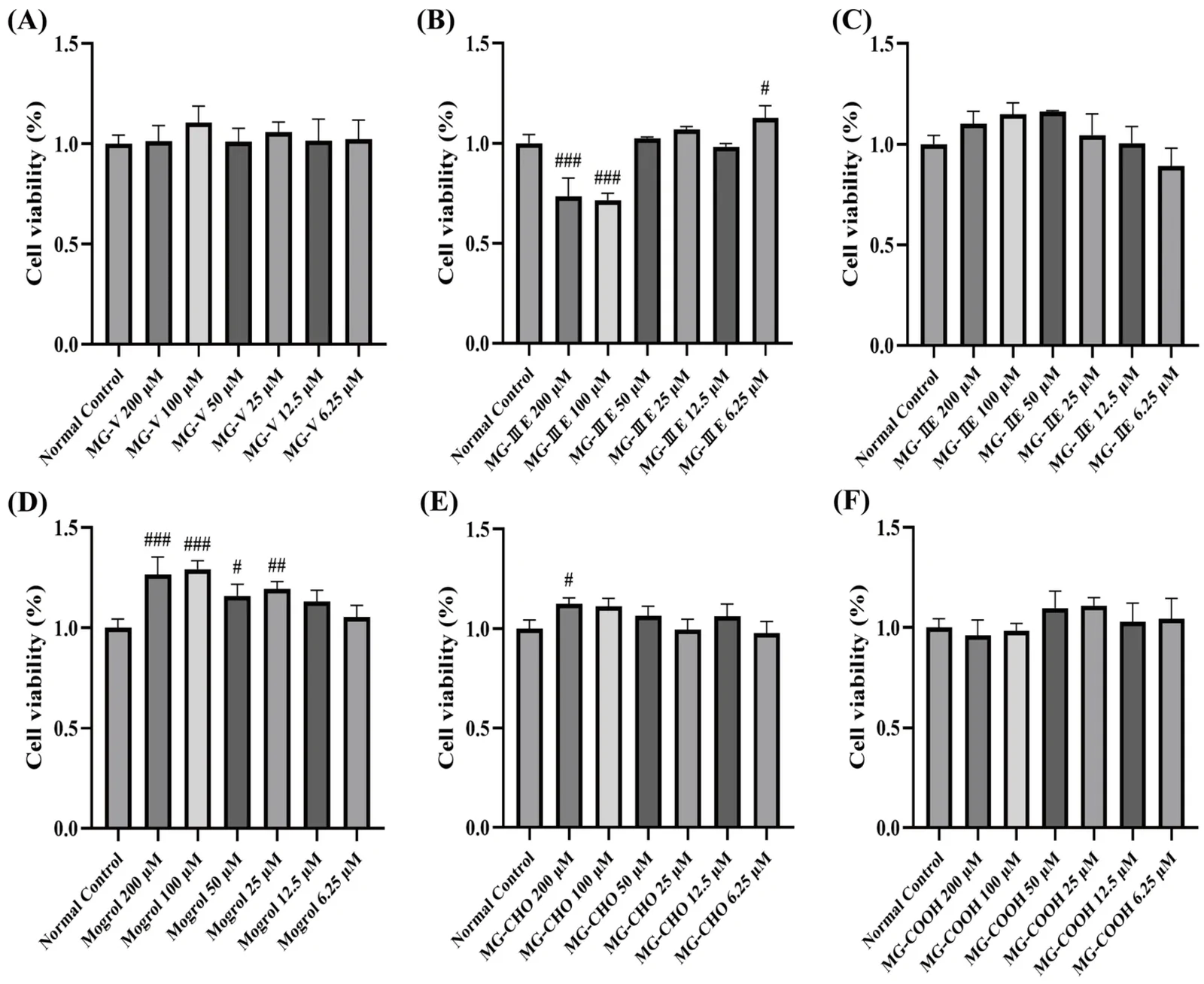
Effects of MG-COOH, MG-CHO, and mogrosides on the viability of MH-S cells. (**A**–**F**) Bar charts showing the effects of different concentrations of MG-V, MG-IIIE, MG-IIE, mogrol, MG-CHO, and MG-COOH on cell viability. Data are presented as mean ± SD (*n* = 3 independent biological replicates per group). ^#^ *p* < 0.05, ^##^ *p* < 0.01, ^###^ *p* < 0.001 vs. normal control.

To evaluate the potential cytotoxicity of MG-COOH, MG-CHO, and mogrosides, we assessed the viability of murine alveolar macrophage MH-S cells following treatment with these compounds at concentrations ranging from 200 μM to 6.25 μM using the CCK-8 assay (Figure 2). Compared with the normal control group, MG-CHO, MG-COOH, and mogrosides did not significantly affect cell viability at concentrations between 6.25 μM and 50 μM. Notably, MG-IIIE markedly reduced cell viability at 100–200 μM (Figure 2B), whereas the other compounds, including MG-CHO and MG-COOH, showed no apparent cytotoxicity effect at 100 μM and 200 μM. Therefore, concentrations of 25 μM and 50 μM were selected for subsequent mechanistic studies.

### 2.3. MG-COOH, MG-CHO and Mogrosides Inhibit LPS-Induced Nitric Oxide (NO) Secretion in MH-S Cells

**Figure 3:**
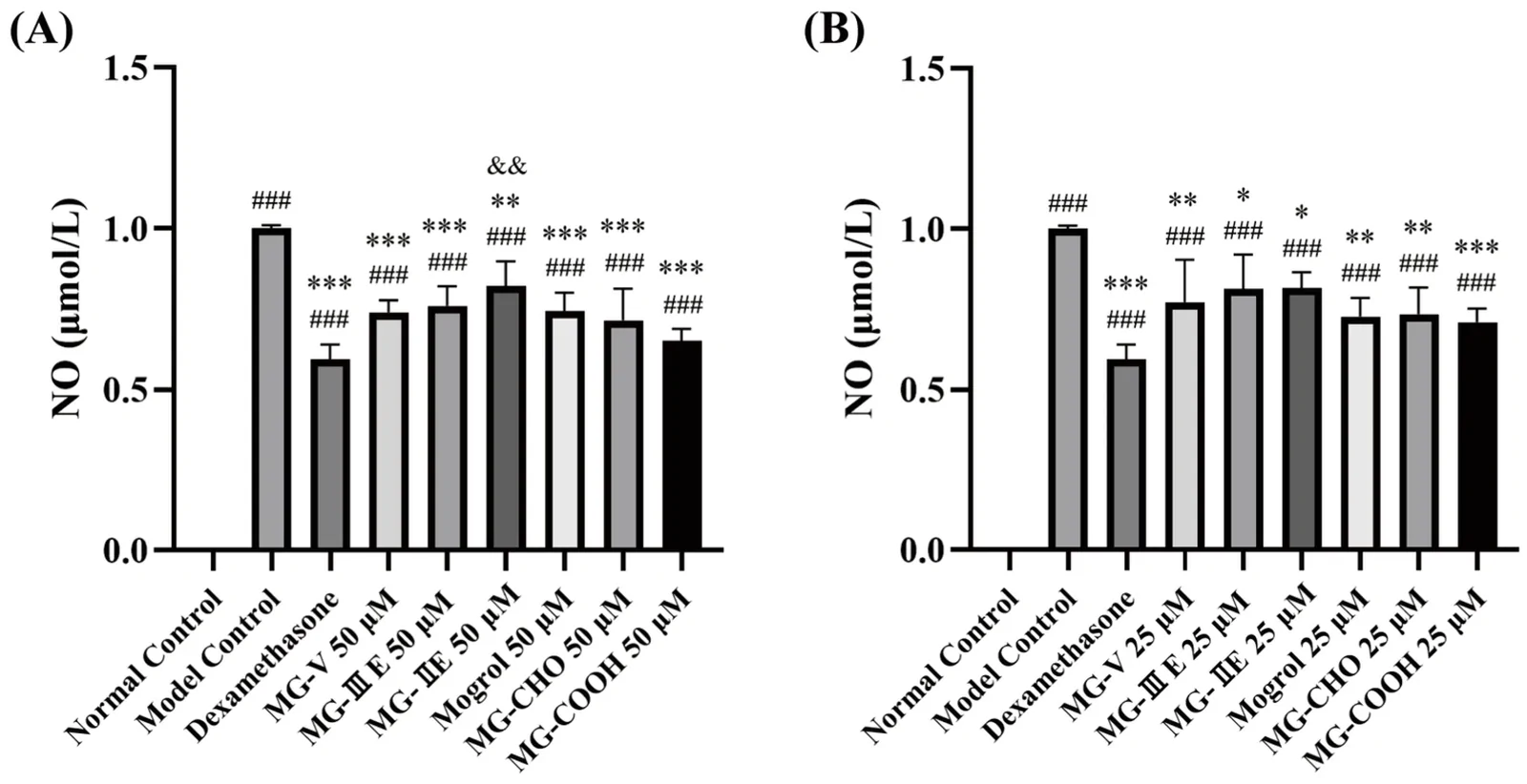
MG-COOH, MG-CHO and mogrosides reduce LPS-induced NO secretion in MH-S cells. NO levels in MH-S cells following pretreatment with MG-V, MG-IIIE, MG-IIE, mogrol, MG-CHO, and MG-COOH at concentrations of 50 μM (**A**), 25 μM (**B**), respectively. Data are presented as mean ± SD (*n* = 3 independent biological replicates per group). ^###^ *p* < 0.001 vs. normal control. * *p* < 0.05, ** *p* < 0.01, *** *p* < 0.001 vs. model control. ^&&^ *p* < 0.01 vs. MG-COOH.

Compared with the normal control group, NO production was significantly elevated in the model control group (*p* < 0.001). In contrast, pretreatment with MG-COOH, MG-CHO or mogrosides markedly suppressed LPS-induced NO release. Notably, MH-S cells pretreated with 25 μM or 50 μM MG-COOH showed significantly lower NO levels than those in the model control group (*p* < 0.001). Moreover, MG-COOH tended to reduce NO release numerically greater than the other mogrosides, although this difference did not reach statistical significance (MG-COOH vs. MG-V, MG-IIIE, MG-IIE, mogrol, and MG-CHO: *p* = 0.288, 0.141, 0.009, 0.262, 0.621, at 50 μM. Figure 3). Collectively, these results indicate that MG-COOH, MG-CHO, and mogrosides exert anti-inflammatory effects on LPS-induced inflammatory responses in macrophages.

### 2.4. MG-COOH, MG-CHO, and Mogrosides Reduce LPS-Induced Secretion of TNF-α, IL-6, and IL-1β in MH-S Cells

**Figure 4:**
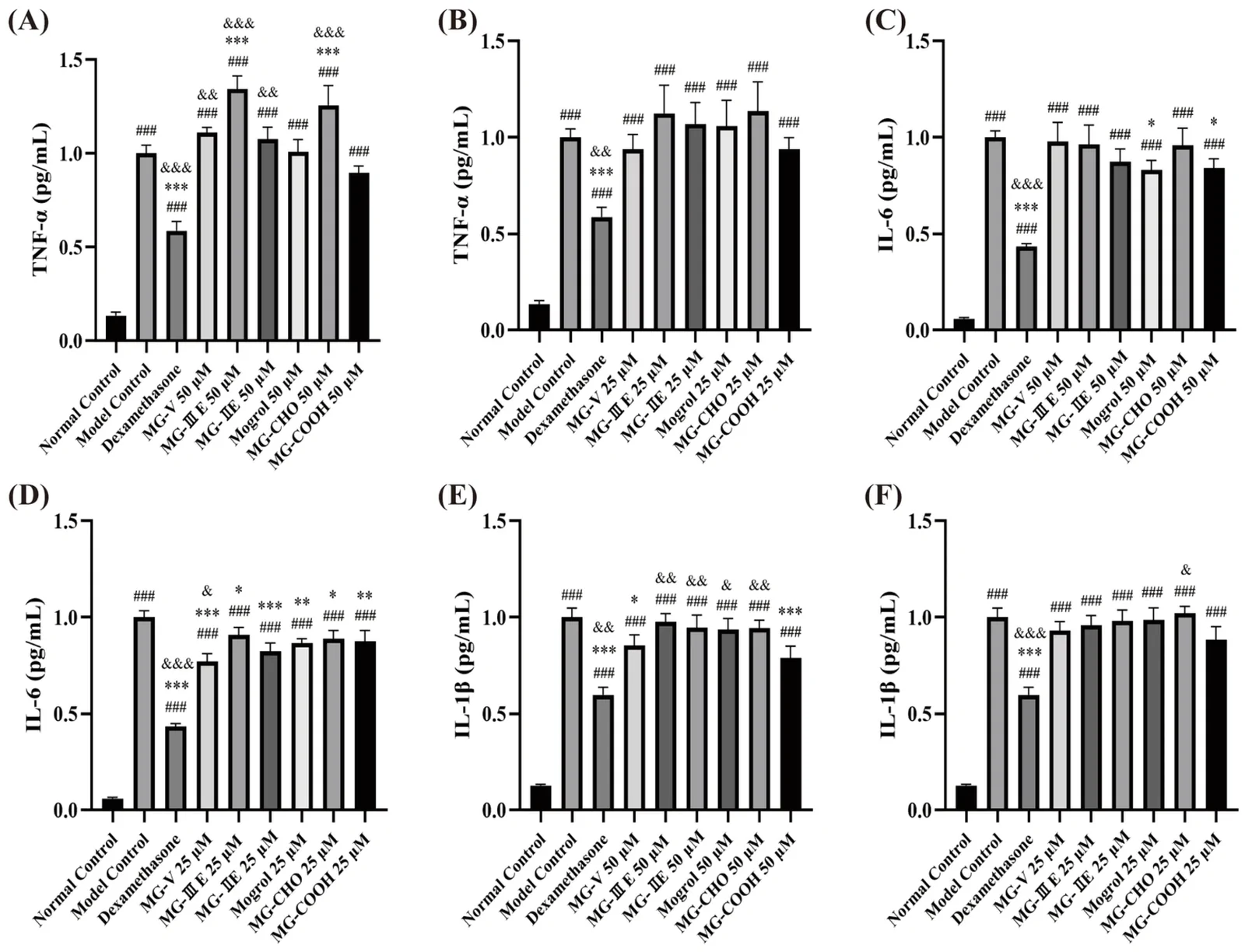
MG-COOH, MG-CHO, and mogrosides decrease LPS-induced release of TNF-α, IL-6 and IL-1β in MH-S cells. (**A**,**B**) TNF-α levels. (**C**,**D**) IL-6 levels. (**E**,**F**) IL-1β levels. Data are presented as mean ± SD (*n* = 3 independent biological replicates per group). ^###^ *p* < 0.001 vs. normal control. * *p* < 0.05, ** *p* < 0.01, *** *p* < 0.001 vs. model control. ^&^ *p* < 0.05, ^&&^ *p* < 0.01, ^&&&^ *p* < 0.001 vs. MG-COOH.

LPS stimulation significantly elevated TNF-α secretion in the model control group relative to the normal control group (*p* < 0.001). At concentrations of 25 μM and 50 μM, MG-COOH reduced TNF-α production, although the effect did not reach statistical significance (*p* > 0.05), whereas the other mogrosides and MG-CHO showed no significant inhibitory effect on TNF-α secretion (Figure 4A,B). Compared with the normal control group, IL-6 secretion was significantly elevated in the model control group (*p* < 0.001). At 25 μM, MG-COOH, MG-CHO and all tested mogrosides significantly reduced the IL-6 levels (*p* < 0.05 or *p* < 0.01, Figure 4C,D). Additionally, IL-1β levels in the supernatants of the model control group were significantly higher than those in the normal control group (*p* < 0.001). Pretreatment with MG-COOH at 25 μM decreased the IL-1β levels slightly, whereas both MG-V and MG-COOH at 50 μM significantly suppressed IL-1β secretion (*p* < 0.05 or *p* < 0.001). Of note, MG-COOH intervention led to markedly less TNF-α and IL-1β release than most of the other tested mogrosides at 50 μM (MG-COOH vs. MG-V, MG-IIIE, MG-IIE, mogrol, and MG-CHO: TNF-α: *p* = 0.002, 0.0001, 0.0089, 0.1534, and 0.0001; IL-1β: *p* = 0.4874, 0.0015, 0.007, 0.0125, and 0.0082.) (Figure 4A,E). These results demonstrate that MG-COOH at 50 μM appears more effective than the tested mogrosides in suppressing the release of pro-inflammatory cytokines such as TNF-α and IL-1β.

### 2.5. MG-COOH and Mogrosides Alleviate LPS-Induced ALI in Mice

**Figure 5:**
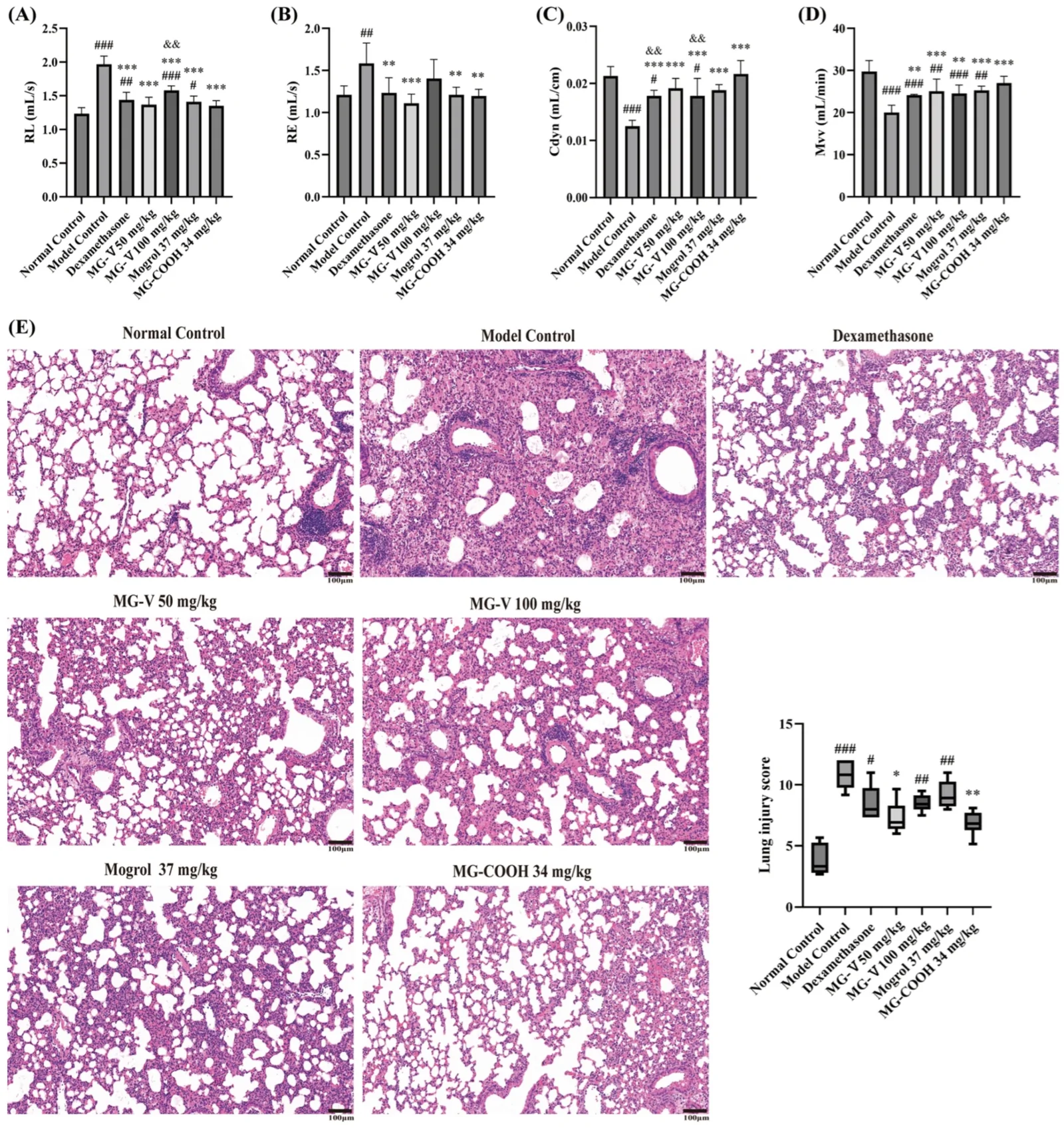
MG-COOH and mogrosides alleviate LPS-induced ALI in mice. (**A**–**D**) RL, RE, Cdyn, and Mvv in pulmonary function assessment (*n* = 6 per group). Data are presented as mean ± SD. (**E**) Representative pictures of H&E staining and statistical analysis of lung injury scores (*n* = 5 for Model control group; *n* = 6 for other groups). Magnification: 20×. Lung injury scores are presented as box-and-whisker plots. Each box denotes the median and the lower and upper quartiles, and the whiskers indicate the minimum and maximum values. ^#^ *p* < 0.05, ^##^ *p* < 0.01, ^###^ *p* < 0.001 vs. normal control, * *p* < 0.05, ** *p* < 0.01, *** *p* < 0.001 vs. model control. ^&&^ *p* < 0.01 vs. MG-COOH 34 mg/kg.

An ALI mouse model was established through intratracheal LPS instillation. A total of 24 h after instillation, the model control mice exhibited significantly elevated inspiratory resistance (RL) and expiratory resistance (RE) (*p* < 0.05), whereas dynamic lung compliance (Cdyn) and maximum voluntary ventilation (Mvv) were markedly decreased compared with the normal control mice (*p* < 0.001, Figure 5A–D). In contrast, pretreatment with MG-COOH or mogrosides significantly reversed these alterations. Notably, the mice pretreated with MG-COOH exhibited a more pronounced reduction in RL (*p* = 0.0013) and a marked elevation in Cdyn (*p* = 0.0048) compared to the MG-V mice.

Lung morphological alterations were assessed using hematoxylin and eosin (H&E) staining (Figure 5E). In the normal control group, the alveolar septa were slender and evenly arranged, with an intact alveolar structure. In contrast, the model control group exhibited marked alveolar septal thickening, massive infiltration of inflammatory cells in the alveolar spaces and pulmonary interstitium, deformed and ruptured alveoli, and disrupted alveolar integrity. The Kruskal–Wallis test revealed a significant difference in the distribution of lung injury scores among the experimental groups (H = 28.697, df = 6, *p* = 0.001). Compared with the model control group, mice pretreated with MG-COOH or MG-V showed substantially alleviated pulmonary morphological damage, reduced inflammatory cell infiltration, restored alveolar structural integrity, and the lung injury scores were significantly reduced (Model vs. MG-COOH and MG-V: *p* = 0.007 and 0.023). Notably, the lung injury scores in the MG-COOH group was numerically lower relative to the MG-V and mogrol groups (*p* = 0.398 and 0.236). Collectively, MG-COOH exhibited a pulmonary protective effect similar to that of mogrosides in LPS-induced ALI mice.

### 2.6. MG-COOH and Mogrosides Inhibit Inflammatory Cytokine Secretion and Oxidative Stress in the Lungs of ALI Mice

**Figure 6:**
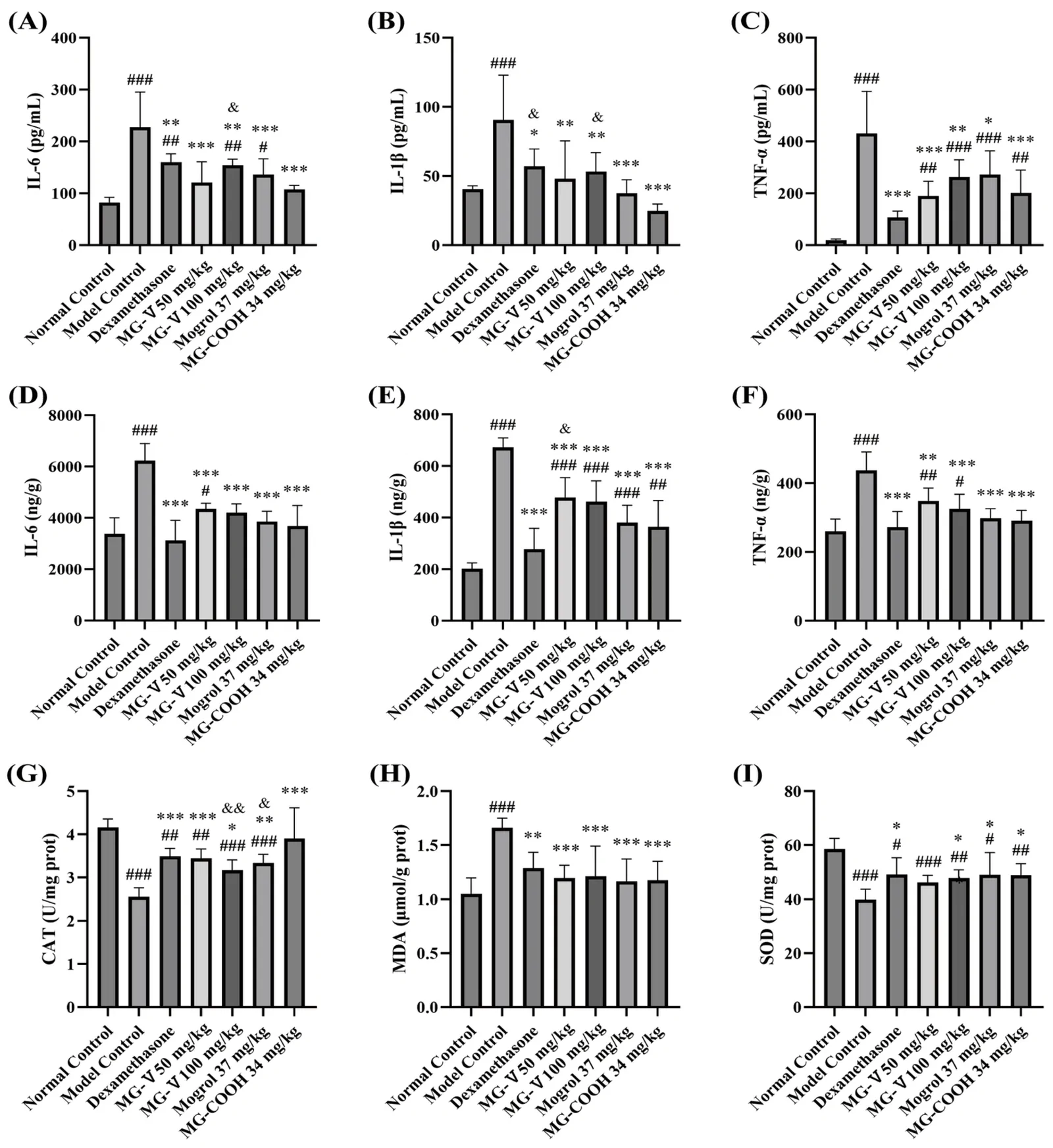
MG-COOH and mogrosides inhibit LPS-induced inflammatory cytokine secretion and oxidative stress in the lungs of ALI mice. (**A**–**C**) IL-6, IL-1β, and TNF-α levels in BALF. (**D**–**F**) IL-6, IL-1β, and TNF-α levels in lungs. (**G**–**I**) MDA, SOD, and CAT levels in lungs. Data are presented as mean ± SD (*n* = 6 per group). ^#^ *p* < 0.05, ^##^ *p* < 0.01, ^###^ *p* < 0.001 vs. normal control, * *p* < 0.05, ** *p* < 0.01, *** *p* < 0.001 vs. model control. ^&^ *p* < 0.05, ^&&^ *p* < 0.01 vs. MG-COOH 34 mg/kg.

The TNF-α, IL-6, and IL-1β levels in BALF were measured by ELISA. Compared with the normal control mice, bronchoalveolar lavage fluid (BALF) cytokine levels were significantly elevated in the model control mice (*p* < 0.001). In contrast, pretreatment with MG-COOH or mogrosides significantly decreased their levels (*p* < 0.05). Notably, the inhibitory effect of MG-COOH on BALF cytokines reached approximately 53%, which was numerically greater than the reductions observed in the groups receiving other mogrosides (Figure 6A–C). Similarly, the measurement of cytokine levels in lung tissues revealed that both MG-COOH and mogrosides significantly suppressed TNF-α, IL-6, and IL-1β (Figure 6D–F). Of note, although the inhibitory effect of MG-COOH on lung TNF-α (33.36% reduction), IL-6 (40.91% reduction), and IL-1β (44.77% reduction) was weaker than its suppression of these cytokines in BALF, it nevertheless displayed numerically greater suppression of pulmonary cytokines than other mogrosides. Regarding oxidative stress status, the model control mice showed a significant increase in MDA content and decreases in SOD and CAT activities compared with the normal control mice (*p* < 0.001). Following pretreatment with MG-COOH or mogrosides, pulmonary MDA levels were markedly reduced, whereas SOD and CAT activities were significantly increased (*p* < 0.05, Figure 6G–I).

### 2.7. Transcriptomic Analysis

**Figure 7:**
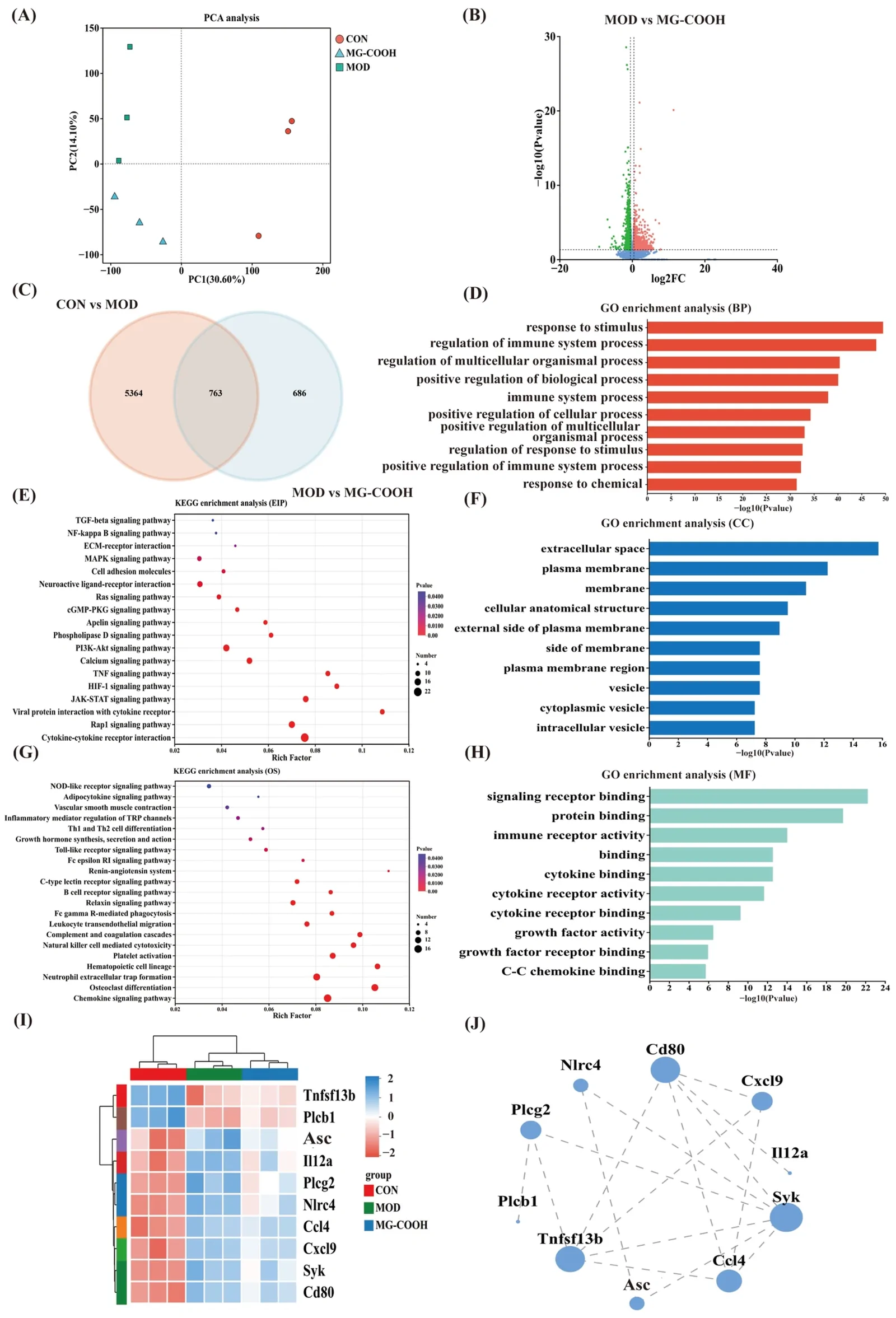
Transcriptomic analysis of the exploratory gene sets (DEGs identification:|log2^FC^| > 0.5, and nominal *p*-value < 0.05). (**A**) PCA analysis (Proportion of Variance: PC1: 30.60%; PC2: 14.10%). (**B**) Volcano plot of differentially expressed genes (DEGs). Green represents downregulated genes, red represents upregulated genes, and blue represents genes with no significant difference. (**C**) Venn diagram of DEGs obtained from pairwise comparisons among the three groups. (**D**,**F**,**H**) GO enrichment histogram of Biological Process (BP), Cellular Component (CC) and Molecular Function (MF) (nominal *p*-value < 0.05). (**E**,**G**) KEGG pathway enrichment bubble diagrams of Environmental Information Processing (EIP) and Organismal Systems (OS) (nominal *p*-value < 0.05). (**I**,**J**) Heatmap and gene network diagram of all DEGs involved in the TLR, NOD, and NF-κB signaling pathways.

RNA-Seq was conducted on the lung tissues of ALI mice to elucidate the molecular mechanisms mediating the protective action of MG-COOH against ALI. Principal component analysis (PCA) demonstrated a clear separation among the three groups, with samples from the MG-COOH group clustering more closely with those of the normal control group, indicating its beneficial protective effect (Figure 7A).

To reconcile false-positive control with retained biological sensitivity, differentially expressed genes (DEGs) were identified under two criteria: an FDR-controlled criterion (|log2^FoldChange (FC)^| > 0.5, BH-adjusted *p* < 0.05), shown in Supplementary Figure S4; and an exploratory criterion (|log2^FC^| > 0.5, nominal *p* < 0.05), shown in Figure 7.

Using the threshold criteria of |log2 ^FC^| > 0.5, and BH-adjusted *p* < 0.05, a total of 5386 DEGs were identified between the model and normal control groups, including 2785 upregulated and 2601 downregulated genes. Comparison between the MG-COOH pretreatment and model control groups identified 536 DEGs, of which 198 were upregulated and 338 were downregulated (Supplementary Figure S4B). Among the 5386 DEGs altered in the model control group, 344 genes displayed a notable restoration toward normal levels following MG-COOH pretreatment (Supplementary Figure S4C). Based on the GRCm39 reference genome, only 93 out of the 344 DEGs were annotated as inflammation-/immune-related and were subjected to subsequent GO (Gene Ontology) and KEGG (Kyoto Encyclopedia of Genes and Genomes) enrichment analyses. Functional enrichment results revealed that these MG-COOH-reverted genes were predominantly enriched in the HIF-1 and TNF signaling pathways (Supplementary Figure S4D–H).

Using an exploratory threshold criteria of |log2^FC^| > 0.5, and nominal *p*-value < 0.05, a total of 6127 DEGs were identified between the model control and normal control groups, including 3231 upregulated and 2896 downregulated genes. Comparison between the MG-COOH and model control groups identified 1449 DEGs, of which 778 were upregulated and 671 were downregulated (Figure 7B). Among the 6127 DEGs altered in the model control group, 763 genes displayed a notable restoration toward normal levels following MG-COOH pretreatment (Figure 7C). Based on the GRCm39 reference genome, 220 out of the 763 DEGs were annotated as inflammation-/immune-related and were subjected to subsequent GO and KEGG enrichment analyses. Functional enrichment results revealed that these MG-COOH-reverted genes were predominantly enriched in the HIF-1, JAK-STAT, MAPK, PI3K-Akt, NF-κB, NOD-like receptor, and Toll-like receptor signaling pathways (Figure 7D–H). In particular, genes associated with the NOD-like receptor and Toll-like receptor signaling pathways exhibited decreased transcript abundance following MG-COOH pretreatment (Figure 7I). However, NOD-like receptor signaling pathway genes were detected exclusively in the exploratory gene set identified by nominal *p*-value < 0.05 and were absent from the FDR-controlled gene set (Figure 7G and Supplementary Figure S4G).

### 2.8. MG-COOH Pretreatment Reduced NLRP3 Inflammasome-Related Inflammatory Responses in the Lung of ALI Mice

**Figure 8:**
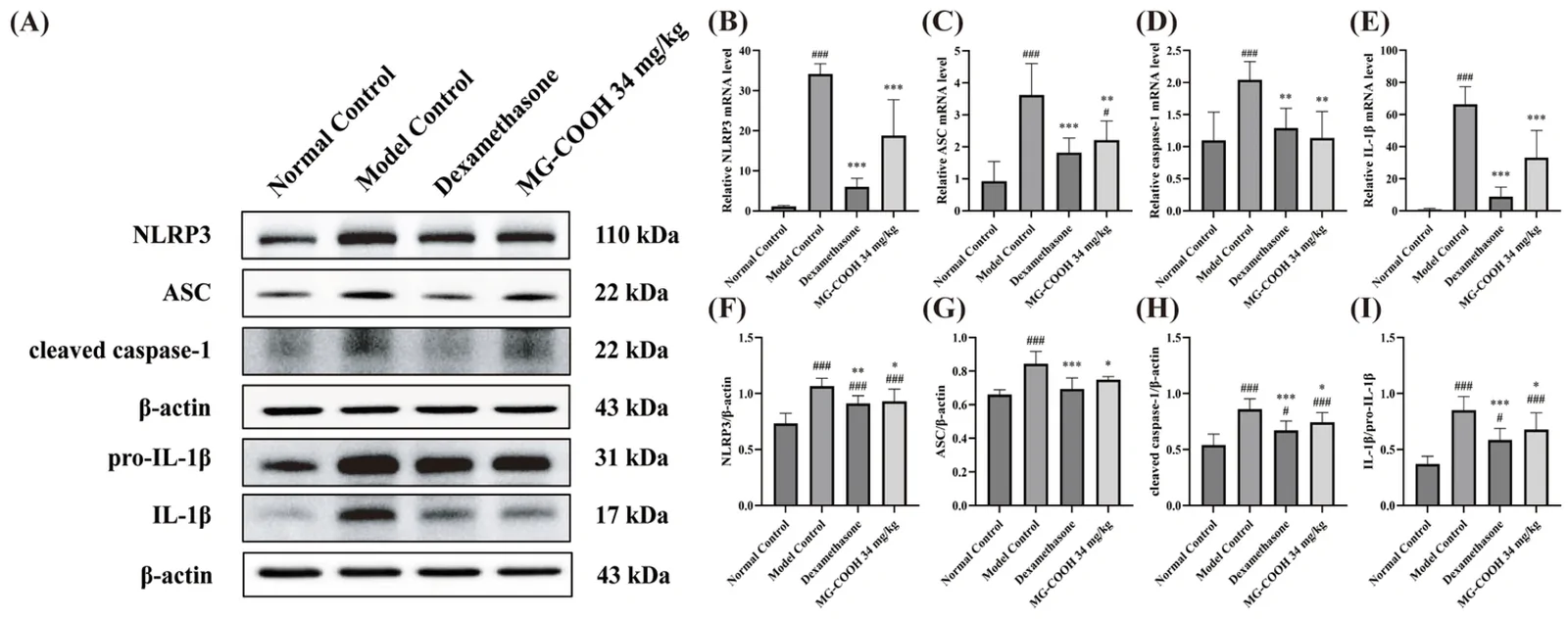
MG-COOH pretreatment reduced NLRP3 inflammasome-related inflammatory responses in the lung of ALI mice. (**A**) Western blot bands of NLRP3, ASC, cleaved caspase-1, IL-1β, and pro-IL-1β. (**B**–**E**) The mRNA expression of NLRP3, ASC, caspase-1, and IL-1β in lungs (*n* = 6 per group). (**F**–**I**) Statistical histogram of Western blot (n = 8 per group for NLRP3, ASC, and cleaved caspase-1; *n* = 7 per group for IL-1β). Differences in biological replicate numbers arose from post-sampling quality control rather than initial animal allocation. Data are presented as mean ± SD. ^#^ *p* < 0.05, ^###^ *p* < 0.001 vs. normal control, * *p* < 0.05, ** *p* < 0.01, *** *p* < 0.001 vs. model control.

Quantitative real-time PCR (RT-qPCR) was used to assess the mRNA levels of NLRP3 inflammasome-related genes in lung tissues (Figure 8B–E). Compared with the normal control group, the mRNA expression of NLRP3 (*p* < 0.001), ASC (*p* < 0.001), caspase-1 (*p* < 0.001), and IL-1β (*p* < 0.001) were significantly upregulated in the model control group. In contrast, pretreatment with MG-COOH significantly downregulated the mRNA levels of NLRP3 (*p* < 0.001), ASC (*p* < 0.01), caspase-1 (*p* < 0.01), and IL-1β (*p* < 0.001) compared with the model control group. Meanwhile, Western blot analysis revealed that intratracheal LPS instillation significantly elevated the protein levels of NLRP3 (*p* < 0.001), ASC (*p* < 0.001), cleaved caspase-1 (*p* < 0.001), and IL-1β/pro-IL-1β ratios (*p* < 0.001) relative to the normal control group. Conversely, pretreatment with MG-COOH significantly decreased the protein abundance of NLRP3 (*p* < 0.05), ASC (*p* < 0.05), cleaved caspase-1 (*p* < 0.05), and IL-1β/pro-IL-1β ratios (*p* < 0.05) relative to the model control group (Figure 8F–I). These findings indicate that MG-COOH pretreatment effectively reduced LPS-induced NLRP3 inflammasome-related inflammatory responses in the lung tissues of ALI mice.

## 3. Discussion

ALI pathogenesis involves an excessive inflammatory response, making the suppression of these pathways a key strategy. ALI generally arises from damage-associated molecular patterns (DAMPs), which facilitate the activation of innate immune receptors, the complement system, and inflammasomes [27]. The NF-κB, MAPK, and JAK-STAT pathways represent the major inflammatory signaling cascades activated in ALI. Activation of these inflammatory cascades initiates an intricate regulatory network that drives pro-inflammatory gene expression. Furthermore, NLRP3 inflammasome activation represents a critical event in ALI, operating as a downstream effector of NF-κB, p38-MAPK, and TLR signaling. Upon activation, it drives massive secretion of IL-1β and IL-18, which in turn aggravate pulmonary injury and feed back to reinforce inflammatory signaling [28]. Thus, the NLRP3 inflammasome not only transduces inflammatory signaling, but also drives the secretion of pro-inflammatory cytokines, serving as a key accelerator in ALI pathophysiology. These features make the NLRP3 inflammasome a promising protective target and an early diagnostic biomarker for ALI.

The ongoing investigation into the pharmacological activities of natural products has revealed many small molecule compounds with considerable protective promise for ALI. *Siraitia grosvenorii*, whose potent anti-inflammatory and antioxidant activities stem primarily from its bioactive constituents, mogrosides, have been reported to confer protective effects against ALI [29]. Accumulating evidence indicates that MG-V and MG-IIIE attenuate LPS-induced lung injury in mice, largely through suppressing the secretion of pro-inflammatory cytokines [10,17]. Mogrol, a deglycosylated derivative of mogrosides, has also been shown to ameliorate lung injury associated with severe acute pancreatitis in rats [30]. In the present study, pretreatment with mogrosides, MG-CHO, and MG-COOH markedly reduced the morphological lung injury scores in ALI mice. Moreover, these agents inhibited the secretion of IL-6, IL-1β, and TNF-α, attenuated pulmonary oxidative stress, and restored impaired respiratory function, consistent with in vitro observations. Of note, MG-COOH showed a tendency toward greater efficacy in reducing inflammation, oxidative stress, and LPS-induced lung injury compared with the other mogrosides under the experimental conditions. Nevertheless, the overall advantage of MG-COOH over the other compounds remains to be confirmed. Meanwhile, one limitation of this study is that only MH-S alveolar macrophages were applied for in vitro assays [31,32]. MG-COOH exerted only mild inhibitory effects on cytokine production in MH-S cells, which could not fully recapitulate its robust anti-inflammatory effect in vivo. In LPS-induced ALI, multiple immune cell subsets (e.g., neutrophils) infiltrate lung tissue and contribute largely to pro-inflammatory cytokine release [33]. Thus, the in vivo protective effect of MG-COOH may involve the regulation of diverse immune cells rather than alveolar macrophages alone. Further studies with additional immune cell models are needed to define its primary cellular targets. Moreover, only male mice were used to ensure consistent phenotypic severity of the LPS-induced ALI model and align with most preclinical literature, but potential sex differences in therapeutic response cannot be excluded. The efficacy of MG-COOH in female mice warrants further investigation.

Oxidative stress triggers inflammatory cascades and upregulates pro-inflammatory cytokines and adhesion molecules, all of which represent key pathogenic factors in ALI. The mutual reinforcement of oxidative stress and inflammation damages alveolar epithelial integrity and pulmonary endothelial barrier function, consequently exacerbating pulmonary injury and edema [34]. Excessive ROS production has been shown to drive NLRP3 inflammasome assembly and IL-1β maturation, thus intensifying inflammation and accelerating ALI progression [35]. Conversely, emodin, an active compound derived from *Rheum palmatum* L., protects against ALI by reducing the ROS, MPO, and MDA levels, an effect attributed to attenuated oxidative stress and suppressed NLRP3 expression in LPS-induced lung injury [36,37,38]. In the present study, MG-COOH enhanced the activity of antioxidant enzymes and alleviated oxidative stress in lung tissues, which may represent a mechanism contributing to its protective effects against ALI.

ALI-associated inflammation involves the activation of multiple signaling cascades, including NF-κB, AMPK, JAK-STAT, and PI3K-Akt [28]. MG-V has been reported to suppress pulmonary inflammation via the NF-κB and JAK-STAT pathways [39], while MG-IIIE exerts anti-inflammatory effects in LPS-induced ALI through the AMPK-dependent downregulation of TLR4/MAPK/NF-κB signaling [10]. In the present study, two criteria were employed for DEG selection in the transcriptomic analysis. The first criterion, |log2^FC^| > 0.5 with BH-adjusted *p* < 0.05, represents the FDR-based approach commonly adopted in transcriptomic research. Under this stringent threshold, however, inflammation-related DEGs were enriched in only 5 signaling pathways, including HIF-1, TNF, and several genes previously implicated in the anti-ALI effects of mogrosides were not captured. To obtain a broader set of DEGs, |log2^FC^| > 0.5 with nominal *p* < 0.05, was applied as an exploratory criterion. This approach revealed that inflammation-related genes enriched in pathways such as HIF-1, JAK-STAT, MAPK, PI3K-Akt, and NF-κB exhibited reduced transcript abundance in the MG-COOH-pretreated group, consistent with findings from other studies on mogrosides.

The divergence between the two criteria suggests that MG-COOH induces modest, coordinated transcriptional changes rather than large alterations in a few genes [40]. Under strict FDR control, the number of drug-responsive genes and enriched pathways was markedly reduced, suggesting that FDR control preferentially filters out these small-magnitude, multi-gene signals. We therefore used nominal *p* < 0.05 with |log2^FC^| > 0.5 for explicitly exploratory, hypothesis-generating enrichment, which may include false-positive genes. While this relatively lenient threshold may capture genes with subtle expression changes, it aligns with the modest, multi-gene coordinated pharmacological effect of the compound. Importantly, the conclusions of this study are not drawn exclusively from transcriptomic bioinformatics, but are corroborated by consistent downstream biochemical and functional experimental data. Although the direct molecular sensor of MG-COOH that triggers reduced inflammatory responses remains uncharacterized in the current study, these results indicate that the oxidized mogrol derivative shares similar mechanisms of action with natural mogrosides in anti-ALI. However, further receptor-binding or molecular interaction experiments are needed to verify these hypothetical candidates.

NF-κB serves as a critical early-phase transcription factor that is activated by LPS, stimulating pro-inflammatory cytokines in macrophages during ALI [41,42,43]. Activated NF-κB promotes the transcription of NLRP3, ASC, and IL-1β, further amplifying the inflammatory cascade. Thus, beyond NF-κB pathway inhibition, targeting the NLRP3 inflammasome has emerged as an additional and valuable strategy for protection against ALI [44]. Genetic deletion of NLRP3 markedly attenuated lung injury and lowered IL-1β and IL-18 concentrations in both BALF and lung tissue in a septic mouse model of ventilator-induced lung injury [43,45]. In addition, attenuation of the TLR4/NF-κB and NLRP3 signaling pathways has been identified as a key mechanism by which *P. tenuifolia* extract reduces the release of inflammatory cytokines and attenuates the inflammatory response in ALI [46]. In A-549 cells, *Apium graveolens* L. similarly protected against ALI through the inhibition of NF-κB and NLRP3 inflammasome signaling [47]. Notably, MG-COOH suppressed the NOD-like receptor signaling pathway in the present transcriptomic analysis, and concurrently downregulated both the mRNA and protein levels of NLRP3, ASC, and caspase-1 in lung tissues of ALI mice. This effect on the NLRP3 cascade could account for the reduced secretion of inflammatory cytokines and the subsequent amelioration of ALI. Collectively, the present results provide novel insights into the molecular mechanisms by which MG-COOH exerts its anti-ALI actions.

Nevertheless, the upstream regulatory pathways of the NLRP3 inflammasome were not addressed in this study, and ASC speck formation assays, as well as genetic or pharmacological validation targeting NLRP3, were not performed. It remains unclear whether MG-COOH directly suppresses the assembly and activation of the NLRP3 inflammasome, or whether its effect is indirectly mediated through the modulation of upstream signaling cascades, thereby leading to reduced mRNA expression of NLRP3, ASC, and caspase-1. This issue warrants further investigation in our subsequent studies.

In summary, as an oxidized mogrol derivative, MG-COOH exhibited a tendency to be more effective than the natural mogrosides at equimolar doses in protecting against LPS-induced ALI. It was found to affect inflammation-related signaling cascades, including HIF-1, JAK-STAT, MAPK, PI3K-Akt, and NF-κB in a manner similar to natural mogrosides. More importantly, MG-COOH effectively attenuates NLRP3 inflammasome-associated inflammatory responses and subsequent IL-1β secretion in the lungs, which is considered at least one of the critical mechanisms underlying its protective effects against ALI.

## 4. Materials and Methods

### 4.1. Animals

Six-week-old male BALB/c mice (20 ± 2 g, Slaike Jingda Laboratory Animal Co., Ltd, Hunan, China) were housed in an SPF room at 22 ± 2 °C and 50 ± 5% humidity under a 12-h light/dark cycle with free access to food and water. All mouse experiments adhered to the guiding principles of the Chinese Animal Welfare Committee and were approved by the Animal Ethics Committee of Guangxi University of Chinese Medicine (KS20250000-003).

### 4.2. Reagents

MG-V (HY-NO502, 98.23%) was sourced from MedChemExpress (Monmouth Junction, NJ, USA). MG-IIE (A1174, 99.04%) and MG-IIIE (A0845, 98.76%) were purchased from Must Biotech (Chengdu, China). Mogrol (A0225, 97.17%) was purchased from Plant Origin (Nanjing, China). LPS (L8880) and dexamethasone (D8040) were obtained from Solarbio (Beijing, China). Sodium periodate (S817518) was purchased from Macklin Biotech (Shanghai, China). Sodium dihydrogen phosphate (S102313) and sodium chlorite (S104904) were purchased from Aladdin (Shanghai, China). The oxidized mogrol derivatives MG-CHO and MG-COOH, which are not commercially available, were synthesized from mogrol via selective oxidation.

### 4.3. Synthesis of Oxidized Mogrol Derivatives, MG-CHO and MG-COOH

The oxidized mogrol derivatives MG-CHO and MG-COOH were synthesized with optimized procedures according to the reported method [25,26], and the synthetic routes are illustrated in Figure 1.

#### 4.3.1. Preparation of MG-CHO

Mogrol (0.200 g) was dissolved in a 1:1 mixture of water and acetone (15 mL each) within a 500 mL round-bottom flask. Sodium periodate (0.200 g) was dissolved in a water–acetone mixed solvent, and the resulting oxidant solution was added dropwise to the substrate solution. The mixture was refluxed at 60 °C with magnetic stirring for 20 h, and the reaction progress was monitored using TLC.

Once the reaction mixture reached room temperature, 400 mL of ultrapure water was added, and the system was left to crystallize. The precipitated solid was collected by suction filtration to afford dried intermediate MG-CHO with an overall yield of 78.5%. The crude product was used directly in the next step without further purification. The purity of MG-CHO was determined to be 94.70% (area normalization) by analytical HPLC on a YMC-Pack ODS-A C18 column (250 × 10.0 mm I.D., 5 μm, 12 nm) using methanol (A) and 0.1% TFA in H_2_O (B) as the mobile phase at a flow rate of 1.5 mL/min, with UV detection at 203 nm. The HPLC chromatogram and detailed gradient information are provided in the Supplementary Materials (Supplementary Figure S3E and Supplementary Tables S1 and S2).

MG-CHO (Yield 78.5%, white solid): HRMS (APCI) *m*/*z*: calcd for C_27_H_44_O_3_ [M-H]^−^: 415.3218, found 415.3219. ^1^H NMR (500 MHz, CDCl_3_) δ 9.76 (s, 1H), 5.57 (dd, *J* = 4.9, 3.2 Hz, 1H), 3.93 (dd, *J* = 11.4, 5.3 Hz, 1H), 3.46 (s, 1H), 1.14 (d, *J* = 10.1 Hz, 6H), 1.06 (s, 3H), 0.92 (d, *J* = 6.4 Hz, 3H), 0.88 (s, 3H), 0.83 (s, 3H). ^13^C NMR (126 MHz, CDCl_3_) δ 203.12, 142.01, 121.01, 78.73, 76.46, 50.08, 49.36, 47.21, 43.03, 42.02, 41.11, 40.12, 39.75, 35.72, 35.53, 34.06, 29.15, 28.17, 27.92, 26.39, 25.77, 25.69, 25.42, 24.10, 19.00, 18.38, 16.70.

#### 4.3.2. Preparation of MG-COOH

MG-CHO (0.0635 g) was placed in a 100 mL round-bottom flask, followed by the addition of 3 mL tert-butanol and 0.252 mL 2-methyl-2-butene. Separately, 177.8 mg sodium dihydrogen phosphate (NaH_2_PO_4_) and 101.6 mg sodium chlorite (NaClO_2_) were dissolved in 3 mL water, and the aqueous mixture was added dropwise to the flask. The reaction mixture was stirred at room temperature for 20 h and monitored using TLC.

After the reaction, 30 mL saturated aqueous ammonium chloride solution was added to quench, and the mixture was then allowed to stand for 2 h. Water-insoluble solid precipitated on the flask wall, which was collected by suction filtration and washed with water to afford MG-COOH. The purity of MG-COOH was determined to be 96.76% (area normalization) by analytical HPLC on a YMC-Pack ODS-A C18 column (250 × 10.0 mm I.D., 5 μm, 12 nm) using methanol (A) and 0.1% TFA in H_2_O (B) as the mobile phase at a flow rate of 1.5 mL/min, with UV detection at 203 nm. The HPLC chromatogram and detailed gradient information are provided in the Supplementary Materials (Supplementary Figure S3F and Supplementary Tables S3–S5).

MG-COOH (Yield 95.9%, Pale yellow solid): HRMS (APCI) *m*/*z*: calcd for C_27_H_44_O_4_ [M-H]^−^: 431.3167, found 431.3166. ^1^H NMR (500 MHz, CDCl_3_) δ 5.57 (dd, *J* = 4.9, 3.2 Hz, 1H), 3.93 (dd, *J* = 11.1, 5.3 Hz, 1H), 3.46 (d, *J* = 2.8 Hz, 2H), 1.13 (d, *J* = 9.9 Hz, 6H), 1.06 (s, 3H), 0.93 (d, *J* = 6.5 Hz, 3H), 0.87 (s, 3H), 0.82 (s, 3H). ^13^C NMR (126 MHz, CDCl_3_) δ 178.66, 141.96, 121.04, 78.78, 76.51, 50.03, 49.35, 47.20, 43.04, 42.00, 41.28, 40.08, 39.74, 35.69, 35.52, 34.06, 29.11, 27.84, 27.42, 26.38, 25.77, 25.69, 25.41, 24.10, 19.00, 18.27, 16.69.

### 4.4. Cell Culture

The murine alveolar macrophage cell line MH-S (GNM43, National Collection of Authenticated Cell Cultures, Shanghai, China) was maintained in RPMI-1640 medium with 10% fetal bovine serum, 1% penicillin–streptomycin, and 0.05% β-mercaptoethanol.

### 4.5. Cell Viability Assay

The logarithmic growth phase MH-S cells were incubated with mogrosides, MG-CHO or MG-COOH at concentrations of 200, 100, 50, 25, 12.5, and 6.25 μM for 24 h, followed by cell viability evaluation using the CCK-8 assay. Briefly, 15 μL of CCK-8 solution was added to each well. Following 30 min incubation at 37 °C, the optical density was measured at 450 nm [48].

### 4.6. Determination of NO Concentration

MH-S cells in logarithmic growth phase were seeded in 24-well plates. After 12 h, the cells were then pretreated with mogrosides, MG-CHO or MG-COOH at concentrations of 12.5, 25, or 50 μM, or with 1 μM dexamethasone. After 2 h, the cells were stimulated with 1 μg/mL LPS to induce inflammation; both compounds and LPS were maintained in the culture medium for another 24 h. Finally, 50 μL of cell supernatant was collected and combined with Griess reagent, and the absorbance at 540 nm was measured to determine the NO concentration.

### 4.7. Determination of Inflammatory Cytokines by ELISA

The culture supernatants were collected and centrifuged at 4500 rpm for 15 min at 4 °C. The resulting supernatants were then analyzed for TNF-α, IL-6, and IL-1β levels using commercial ELISA kits (Proteintech Biotech, Wuhan, China), following the manufacturer’s instructions.

### 4.8. Establishment of LPS-Induced ALI in Mice

The BALB/c mice were divided into seven groups using a random number table: normal control, model control, dexamethasone (5 mg/kg), mogroside V low dose (MG-V, 50 mg/kg), MG-V high dose (100 mg/kg), mogrol (37 mg/kg), and MG-COOH (34 mg/kg). The doses of MG-V were selected based on a previous report [49]. Mogrol and MG-COOH were given at doses equimolar to 100 mg/kg MG-V (7.77 × 10^−5^ mol/kg) to enable valid comparisons and ensure comparability among the tested mogrosides. All drug pretreatments were given orally once daily for 7 consecutive days, whereas mice in the normal control and model control groups received ddH_2_O via the same route. Two hours after the final administration, all mice were anesthetized with 3% isoflurane. Intratracheal administration of LPS (5 mg/kg) was performed on all mice excluding the normal control group for the induction of ALI, whereas the normal control mice were instilled with saline [50]. 24 h later, the animals were subjected to pulmonary function assessment before deeply being anesthetized with 5% isoflurane and euthanized. The subsequent analyses were conducted using harvested BALF and lung tissues. The number of animals was determined based on the effect size and variability of the primary endpoint in our pilot experiment and in published LPS-induced ALI studies, with an allowance for attrition [10]. A total of 96 mice were used in this study, with 12 mice assigned to each experimental group. 6 mice per group were used for pulmonary function assessment, BALF collection and oxidative stress measurement; another 6 mice per group were used for H&E staining, inflammatory factor detection, RT-qPCR and Western blot analysis. For the normal control, model control, and MG-COOH groups, an additional 3 mice per group were included for transcriptome sequencing and Western blot analysis of lung tissue, whereas 3 additional mice in the dexamethasone group served as a positive control, with their lung tissues used only for Western blot analysis and not for transcriptome sequencing. These animals were subjected to the same modeling and pretreatment procedures at identical time points to minimize the influence of non-drug-related factors. Histopathological scoring, sample collection, and biochemical assays were performed by investigators blinded to group assignment. Samples were excluded according to pre-established criteria: death during LPS challenge or anesthesia; severe hemolysis in BALF; confirmed protein degradation in tissue; or compromised RNA integrity in lung samples. One sample per group was excluded from the IL-1β Western blot analysis due to protein degradation, resulting in a final sample size of *n* = 7 per group.

### 4.9. Pulmonary Function Assessment

At 24 h after ALI induction, mice were anesthetized with 3% isoflurane. A midline neck incision was made to expose the trachea. Tracheal intubation was then performed, and the intubation tube was connected to an AniRes2005 pulmonary function testing system (Belamb Technology, Sichuan, China). The mice were placed in a plethysmograph chamber to assess respiratory parameters. The ventilator was set at a respiratory rate of 90 breaths/min with an inspiratory-to-expiratory ratio of 2:1, then the RL, RE, Cdyn, and Mvv were measured.

### 4.10. Detection of Inflammatory Cytokines in BALF

After anesthesia and euthanasia, the trachea was exposed via cervical incision. An indwelling needle was inserted into the trachea, and 1.5 mL of PBS was slowly instilled into the lungs in three separate aliquots, followed by gentle aspiration to collect BALF into EP tubes. BALF was centrifuged at 1500 rpm for 10 min at 4 °C, and the supernatant was harvested. TNF-α, IL-6, and IL-1β levels were measured following the ELISA kit instructions (Proteintech Biotech, Wuhan, China).

### 4.11. H&E Staining

After euthanasia, the thoracic cavity was incised to expose the heart, and saline was used to flush the pulmonary vasculature via the left ventricle. The lung was preserved in 4% paraformaldehyde, underwent ethanol dehydration in increasing concentrations, embedded in paraffin, and then sectioned. Following dewaxing and rehydration, sections were stained with H&E (Saiviewer, Wuhan, China). Lung histopathology was evaluated using light microscopy. Histopathological injury was scored based on five features: inflammatory cell infiltration, alveolar exudate/hemorrhage, alveolar wall thickening, alveolar disruption, and hyaline membrane deposition. Each parameter was graded on a scale from 0 to 3 (0 = no injury; 1 = mild injury; 2 = moderate injury; 3 = severe injury). For each animal, 1 lung tissue section was examined, and 3 random non-overlapping fields were evaluated per section. The final total lung injury score was calculated as the sum of scores from the five individual parameters, with a total score ranging from 0 to 15. This scoring system was adapted from a previously validated acute lung injury histopathological scoring system [51].

### 4.12. Detection of Pulmonary Inflammatory Cytokines and Oxidative Stress Indicators

Lung tissues were dissected, weighed, and cryogenically ground with saline at a 1:9 (*w*/*v*) ratio to create 10% tissue homogenates. Following centrifugation of the homogenates at 3000 rpm for 15 min at 4 °C, the supernatants were obtained. TNF-α, IL-6, IL-1β, MDA, SOD, and CAT levels were assayed using commercial kits (Jiancheng, Nanjing, China).

### 4.13. Transcriptome Sequencing

From the normal control, model control, and MG-COOH groups, three lung samples per group were randomly chosen for transcriptomic analysis. The transcriptome was analyzed using the NovaSeq Reagent Kit by Majorbio Bio-Pharm Technology Co., Ltd (Shanghai, China). Briefly, total RNA was extracted from tissues with TRIzol reagent and subjected to quality inspection. Sequencing libraries were constructed using the Illumina Stranded mRNA Prep Kit. DEGs were screened using DESeq2 with two sets of thresholds: (1) |log2^FC^| > 0.5 and BH-adjusted *p*-value < 0.05; and (2) |log2^FC^| > 0.5 and normal *p*-value < 0.05. The corresponding results are reported separately. GO and KEGG enrichment analyses based on the hypergeometric distribution were conducted to identify significantly enriched functional terms.

### 4.14. RT-qPCR Analysis

Lung tissue total RNA was extracted using an RNA extraction kit (LS1040, Promega, Beijing, China) and reverse transcribed into cDNA using a commercial kit (M1682, Thermo, Waltham, MA, USA). RT-qPCR was performed on a real-time detection system using SYBR Green (A25742, Thermo, Waltham, MA, USA) with β-actin as the internal reference gene. The 2^−ΔΔCt^ method was employed to calculate the relative expression levels of target genes. The primer sequences for each gene are listed in Table 1.

### 4.15. Western Blot Analysis

Proteins were extracted from lung samples using a commercial kit (BOSTER, Wuhan, China) and quantified with a BCA assay (Keygen, Jiangsu, China). The protein samples were diluted to a uniform concentration and denatured by boiling at 100 °C. Then, 50 μg of protein was separated by 10% or 12.5% SDS-PAGE and transferred onto PVDF membranes (Sigma-Aldrich, St. Louis, MO, USA). The membranes were blocked with 5% non-fat milk for 2 h and then incubated overnight at 4 °C with primary antibodies against NLRP3 (1:2000, 30109-1-AP) and β-actin (1:2000, 20536-1-AP), which were purchased from Proteintech (Wuhan, China); IL-1β (1:1000, ab254360) and ASC (1:1000, ab309497) were purchased from Abcam (Cambridge, MA, USA); and cleaved caspase-1 (1:1000, 89332S, Cell Signaling Tech, Danvers, MA, USA). After washing, the membranes were incubated with HRP-conjugated secondary antibodies (1:10,000) for 2 h at room temperature. Immunoreactive bands were visualized using an ECL chemiluminescent kit (Beyotime, Shanghai, China), quantified with ImageJ 1.54s (National Institutes of Health, Bethesda, MD, USA), and the relative expression levels of proteins were normalized to β-actin.

### 4.16. Statistical Analysis

Data were analyzed and processed using GraphPad Prism 10.6.1 (San Diego, CA, USA). Normality was checked with the Shapiro–Wilk test. Normally distributed data were analyzed by one-way analysis of variance (ANOVA) followed by Dunnett’s post hoc test for multiple comparisons, and data are presented as mean ± SD. Lung injury score were analyzed using the Kruskal–Wallis test followed by Dunn’s post hoc test, and data are expressed as median and interquartile range. *p* < 0.05 was considered statistically significant.

## Figures and Tables

**Table 1:** The primer sequence list for RT-qPCR.

Gene	Forward Primer	Reverse Primer
ASC	GACGCCATAGATCTCACTGACAA	TGCTCAGCCAGCTCCTGTAA
NLRP3	TCAGGATCTCGCATTGGTTCT	CTGTGGGTCCTTCATCTTTTCAC
IL-1β	CCAGGATGAGGACATGAGCAC	TGTTGTTCATCTCGGAGCCTGTA
caspase-1	TCCGAGGGTTGGAGCTCAAG	CTGGGCAGGCAGCAAATTC
β-actin	CATCCGTAAAGACCTCTATGCCAAC	ATGGAGCCACCGATCCACA

## Data Availability

The original contributions presented in this study are included in the article/Supplementary Materials. Further inquiries can be directed to the corresponding author(s). Data from RNA-Seq is available at the GEO repository under accession GSE347446 (NCBI, USA).

## Supplementary Materials

The following supporting information can be downloaded at: https://www.mdpi.com/article/10.3390/ijms27219584/s1.

## Abbreviations

The following abbreviations are used in this manuscript:

ALI	Acute lung injury
LPS	Lipopolysaccharide
ROS	Reactive oxygen species
RL	Inspiratory resistance
RE	Expiratory resistance
Cdyn	Dynamic lung compliance
Mvv	Maximal voluntary ventilation
BALF	Bronchoalveolar lavage fluid
PCA	Principal component analysis
GO	Gene Ontology
KEGG	Kyoto Encyclopedia of Genes and Genomes
BP	Biological process
MF	Molecular function
EIP	Environmental information processing
OS	Organismal systems
RT-qPCR	Reverse transcription quantitative polymerase chain reaction
