# Supplementary material for: An Oxidized Mogrol Derivative Alleviates LPS-Induced Acute Lung Injury in Association with Reduced NLRP3 Inflammasome-Associated Inflammatory Responses in Mice
Source: Int J Mol Sci. 2026 Oct 27;27(21):9584. doi: 10.3390/ijms27219584 (PMC13763169; doi:10.3390/ijms27219584)
Supplement: Supplementary file 1 [file ijms-27-09584-s001.zip › Supplementary_Material-R4.pdf]

Supplementary Material

Supplementary Figures

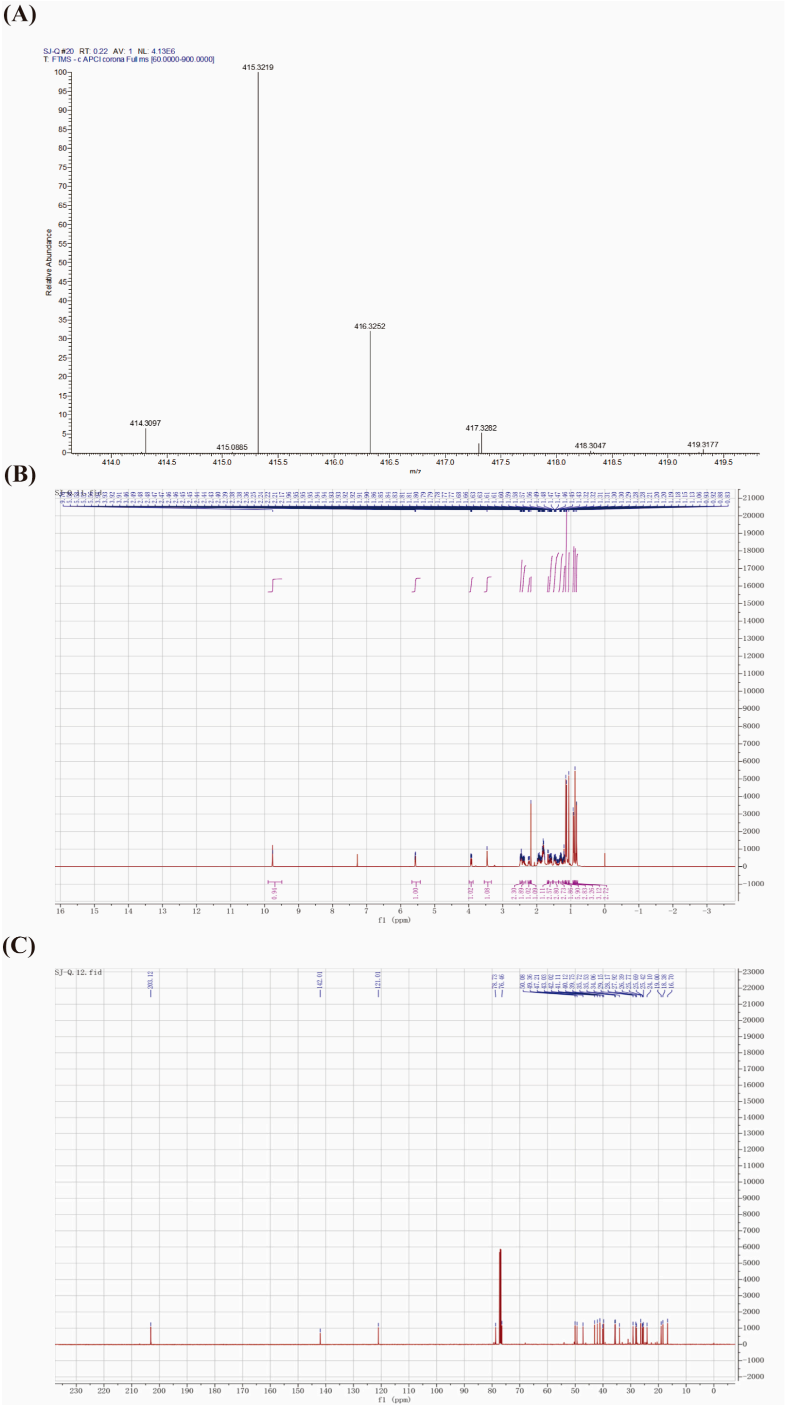

Supplementary Figure S1. Characterization of MG-CHO. **(A)** Mass spectrum. **(B)** ^{1}H NMR. **(C)** ^{13}C NMR.

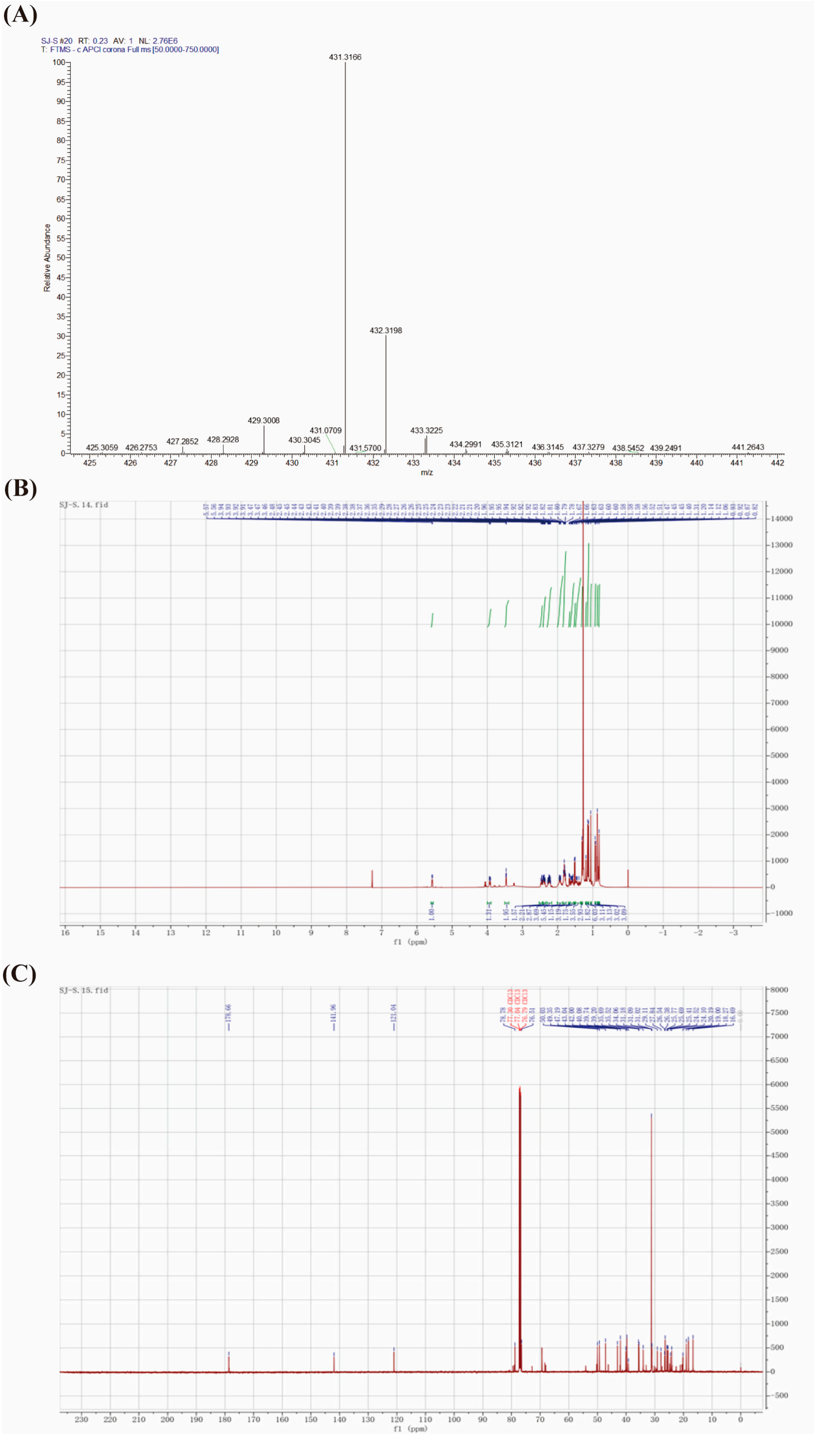

Supplementary Figure S2. Characterization of MG-COOH. **(A)** Mass spectrum. **(B)** ^{1}H NMR. **(C)** ^{13}C NMR.

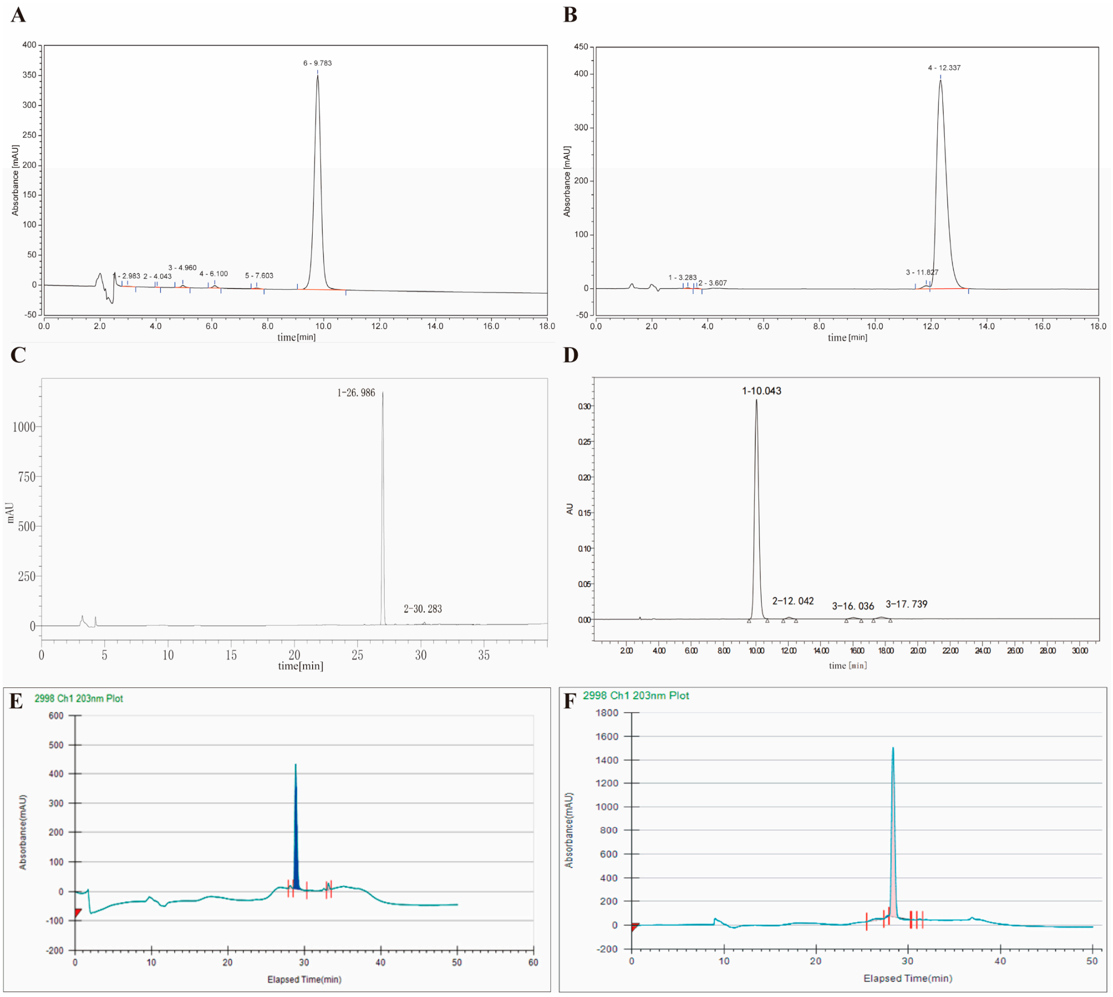

Supplementary Figure S3. HPLC chromatogram of mogrosides. **(A)** MG-V 98.23%. **(B)** MG-IIIE 98.76%. **(C)** MG-IIE 99.04%. **(D)** MG-OH 97.17%. **(E)** MG-CHO 94.70%. **(F)** MG-COOH 96.76%.

Supplementary Transcriptomic Analysis

Using the threshold criteria of |log2$^{\text{FoldChange (FC)}}$| > 0.5, and BH-adjusted $p < 0.05$, a total of 5,386 differentially expressed genes (DEGs) were identified between the model and normal control groups, including 2,785 upregulated and 2,601 downregulated genes. Comparison between the MG-COOH pretreatment and model control groups identified 536 DEGs, of which 198 were upregulated and 338 were downregulated (Supplementary Figure 4B). Among the 5,386 DEGs altered in the model control group, 344 genes displayed a notable restoration toward normal transcript levels following MG-COOH pretreatment (Supplementary Figure 4C). Based on the GRCm39 reference genome, 93 of the 344 DEGs were annotated as inflammation-/immune-related and were subjected to subsequent GO (Gene Ontology) and KEGG (Kyoto Encyclopedia of Genes and Genomes) enrichment analyses. Functional enrichment results revealed that these MG-COOH-reverted genes were predominantly enriched in HIF-1 and TNF signaling pathways (Supplementary Figures 4D-H). Under FDR control, only a limited number of drug-responsive inflammation-related genes were identified, and the enriched signaling pathways were correspondingly restricted.

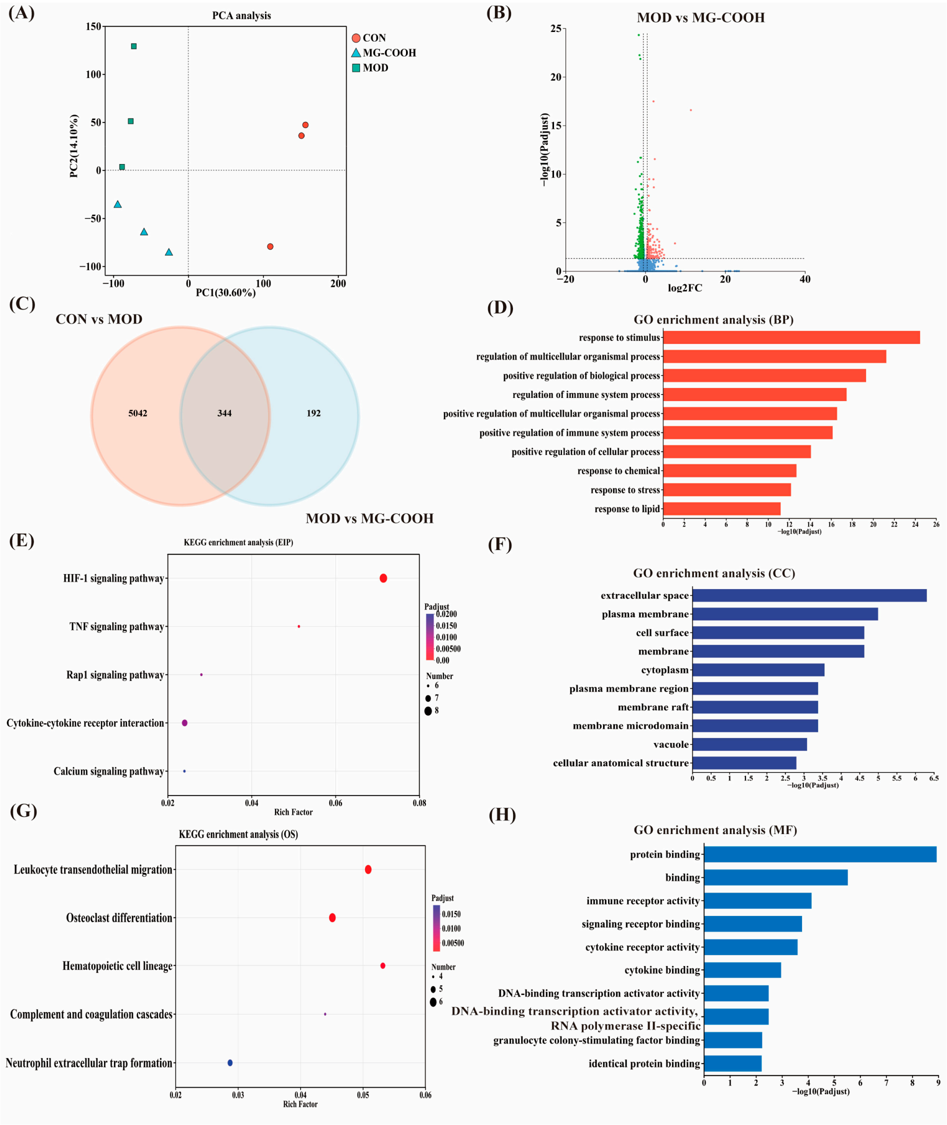

Supplementary Figure S4. Transcriptomic analysis of the FDR-controlled gene sets (DEGs

identification: $|\log 2^{FC}| > 0.5$, and BH-adjusted $p < 0.05$). (**A**) PCA analysis (Proportion of Variance: PC1: 30.60%; PC2: 14.10%). (**B**) Volcano plot of differentially expressed genes (DEGs). Green represents downregulated genes, red represents upregulated genes, and blue represents genes with no significant difference. (**C**) Venn diagram of DEGs obtained from pairwise comparisons among the three groups. (**D,F,H**) GO enrichment histogram of Biological Process (BP), Cellular Component (CC) and Molecular Function (MF) (BH-adjusted $p < 0.05$). (**E,G**) KEGG pathway enrichment bubble diagrams of Environmental Information Processing (EIP) and Organismal Systems (OS) (BH-adjusted $p < 0.05$).

Supplementary Tables

Product Name	: MG-CHO
Catalog ID	:GT-M10572
Column	:YMC-Pack ODS-A, AA12S05-2510WT, C18, (250x10.0ml.D, S-5μm,12 nm)
Buffer	: A: Methanol B: 0.1% TFA in H_2O
Flow rate	:1.5ml/min
Wavelength	:203nm

Table S1. HPLC conditions for MG-CHO analysis.

Peak Information

Peak No	% Area	Area	Ret. Time	Height	Cap. Factor
1	1.9258	179072.4048	28.13 min	9.8936	28132.3333
2	94.6972	8805710.2845	28.85 min	425.631	28849
3	3.3771	314028.438	33.13 min	22.18	33132.3333

Table S2. Peak information of MG-CHO HPLC.

Product Name	: MG-COOH
Catalog ID	:GT-M10572
Column	:YMC-Pack ODS-A, AA12S05-2510WT, C18, (250x10.0ml.D, S-5μm,12 nm)
Buffer	: A: Methanol B: 0.1% TFA in H_2O
Flow rate	:1.5ml/min
Wavelength	:203nm

Table S3. HPLC conditions for MG-COOH analysis.

Elute gradient:

Time (min)	Flow (ml/min)	Solvent %A	Solvent %B
Initial	1.5	5.0	95.0
0.01	1.5	5.0	95.0
10.00	1.5	95.0	5.0
12.00	1.5	100.0	0.0
20.00	1.5	100.0	0.0
37.00	1.5	5.0	95.0
38.00	1.5	100.0	0.0
50.00	1.5	100.0	0.0

Table S4. HPLC gradient-elution program.

Peak Information

Peak No	% Area	Area	Ret. Time	Height	Cap. Factor
1	2.3557	788790.002	26.74 min	9.6198	26740.6667
2	0.4689	157019.6546	27.8 min	7.4982	27799
3	96.7571	32397930.5711	28.36 min	1432.5132	28357.3333
4	0.0504	16869.2194	30.58 min	1.0284	30582.3333
5	0.3678	123163.5273	31.26 min	8.2846	31257.3333

Table S5. Peak information of MG-COOH HPLC.
